# Supplementary material for: Counting and Correcting Thermodynamically Infeasible Flux Cycles in Genome-Scale Metabolic Networks
Source: Metabolites. 2013 Oct 14;3(4):946–66. doi: 10.3390/metabo3040946 (PMC3937828; doi:10.3390/metabo3040946)
Supplement: Supplementary File 1 — Supplementary (ZIP, 361 KB) [file metabolites-03-00946-s002.zip › metabolites-03-00946-supplementary/metabolites-03-00946-S3.pdf]

Metabolites **2013**, *3*, 946-966; doi:10.3390/metabo3040946

OPEN ACCESS

metabolites

ISSN 2218-1989

www.mdpi.com/journal/metabolites

Article

Supporting Text: Counting and Correcting Thermodynamically Infeasible Flux Cycles in Genome-Scale Metabolic Networks

Daniele De Martino [1,2], Fabrizio Capuani [1], Matteo Mori [1], Andrea De Martino [1,2,3,†,*] and Enzo Marinari [1,2,†,*]

[1] Dipartimento di Fisica, Sapienza Università di Roma, p.le A. Moro 2, Roma 00185, Italy
[2] Center for Life Nano Science@Sapienza, Istituto Italiano di Tecnologia, v. Regina Elena 291, Roma 00151, Italy
[3] CNR-IPCF, UOS di Roma, Dipartimento di Fisica, Sapienza Università di Roma, Roma 00185, Italy

[†] These authors contributed equally to this work.

* Authors to whom correspondence should be addressed; E-Mails: andrea.demartino@roma1.infn.it, enzo.marinari@uniroma1.it; Telephones: +39-06-4991-4394 (A.D.M), +39-06-4991-4363 (E.M.); Fax: +39-06-4957-697 (A.D.M.), +39-06-9725-7050 (E.M.)

Received: 6 August 2013; in revised form: 18 September 2013 / Accepted: 24 September 2013 / Published: 14 October 2013

Abstract: We discuss (a) a toy example showing explicitly that Equation (2) of the main text implies the absence of infeasible cycles and (b) the advantages of using an annealing schedule over a fixed-temperature Monte Carlo to find the global minima of a complex function.

Keywords: supporting text

---

1. $\mu\Omega > 0$ implies absence of infeasible reaction cycles

In this section, we present an example that shows that a feasible flux configuration necessarily satisfies the Equation:

$$\boldsymbol{\mu\Omega} > \mathbf{0} \quad , \tag{1}$$

while an infeasible flux configuration does not. Let us consider the network shown in Figure 1. For such

Figure 1. A toy reaction network with a feasible (**left**) and an infeasible (**right**) flux configuration. With the flux configuration on the left, we can associate three chemical potentials, which must satisfy $\mu_A > \mu_B > \mu_C$, while for the configuration on the right, we cannot.

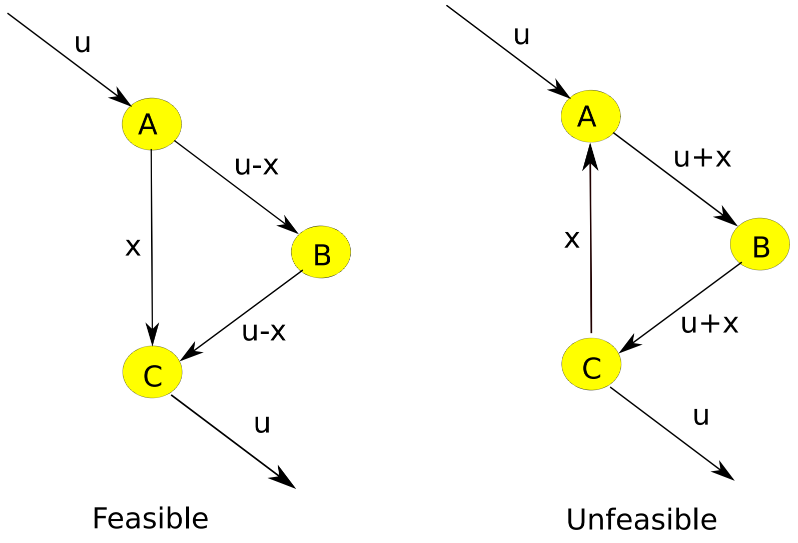

wiring, the internal stoichiometric matrix (without uptakes) reads:

$$\mathbf{S} = \begin{pmatrix} -1 & 0 & -1 \\ 1 & -1 & 0 \\ 0 & 1 & 1 \end{pmatrix} . \tag{2}$$

For the configuration on the left in Figure 1, all fluxes are positive, so that the matrix $\mathbf{\Omega}$, with elements $\Omega_{mr} = -\text{sign}(v'_r)S_{mr}$, becomes:

$$\mathbf{\Omega} = \begin{pmatrix} 1 & 0 & 1 \\ -1 & 1 & 0 \\ 0 & -1 & -1 \end{pmatrix} \tag{3}$$

After defining $\boldsymbol{\mu} = (\mu_A, \mu_B, \mu_C)$, Equation (1) corresponds to the system of inequalities:

$$\begin{cases} \mu_A > \mu_B \\ \mu_B > \mu_C \\ \mu_A > \mu_C \end{cases} , \tag{4}$$

which is satisfied by any chemical potentials, as long as $\mu_A > \mu_B > \mu_C$. If, instead, the flux configurations are given by the right one in Figure 1, the flux connecting A and C have a negative sign, and the matrix $\mathbf{\Omega}$ becomes:

$$\mathbf{\Omega} = \begin{pmatrix} 1 & 0 & -1 \\ -1 & 1 & 0 \\ 0 & -1 & 1 \end{pmatrix} . \tag{5}$$

For this flux configuration, Equation (1) does not have a solution, as can be seen immediately by the corresponding systems of inequalities:

$$\begin{cases} \mu_A > \mu_B \\ \mu_B > \mu_C \\ \mu_A < \mu_C \end{cases} . \tag{6}$$

2. Fixed-Temperature Monte Carlo *Versus* Annealing

When sampling flux configurations with a Monte Carlo (MC) at fixed temperature, the Metropolis Markov Chain can get stuck in one of the (many) local energy minima, i.e. flux patterns with $E > 0$ that are minima with respect to local changes of the configuration. To speed up convergence to the absolute minima with $E = 0$ (i.e., to cycles), one can resort to a convenient temperature scheduling. As an example, we show in Figure 2 the value of E as a function of the number of MC steps for the dynamics simulated on a small (104 reactions) chemical reaction network (taken from [1]), both for a simple Metropolis at fixed temperature $T = 0.25$ and for an annealing schedule in which the system is cooled from $T = 1$ to $T = 0$ and, then, heated back to $T = 1$ repeatedly with a cooling/reheating rate of $|\Delta T| = 10^{-3}$. Annealing finds a minimum in about 10^5 steps, while the basic MC converges after $3 \cdot 10^7$ steps (not shown).

Figure 2. The dynamics of the energy as a function of the number of Monte Carlo steps for the simulated annealing (red) and the fixed temperature scheme (green, $T = 0.25$) for the reaction network of [1]. The figure shows that while at fixed temperature, the Monte Carlo procedure is trapped into a local minimum (and never reaches zero), when supplemented with an annealing schedule, it reaches the global minimum.

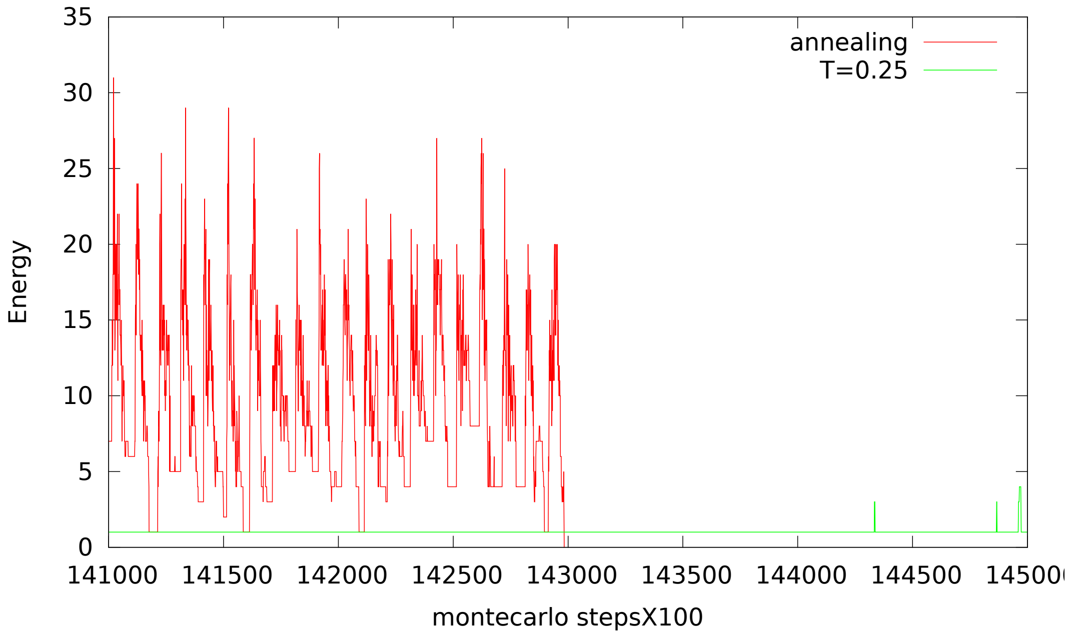

In Figure 3, we display the average MC steps necessary to find all loops as a function of T in two subnetworks of the cerebral_cortex_neuronal tissue network, which are composed of 43 and 23 reactions, respectively. The figure shows that the annealing scheme always requires less MC steps. We note, however, that there can be an optimal temperature, such that the fixed temperature MC reaches the global minimum in a feasible time, but this optimal temperature depends on the network analyzed.

Figure 3. Average Monte Carlo steps necessary to find a loop with a simple metropolis scheme (red lines) as a function of the temperature in two different subnetworks of the cerebral_cortex_neuronal tissue-specific network, of a size of 43 (right) and 23 (left). The optimal T depends on the subnetwork, and the corresponding minimum average time is slightly higher than the one performed by the annealing scheme (green lines).

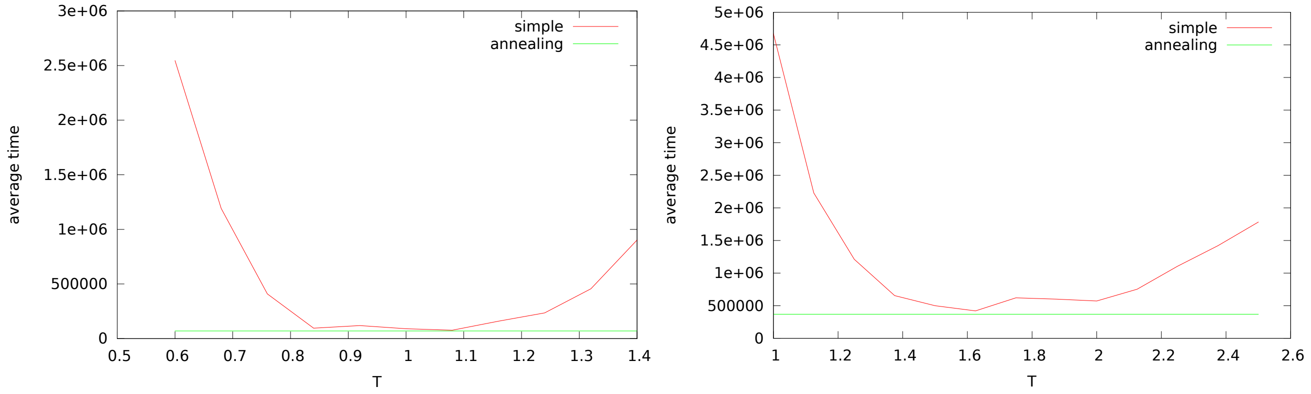

References

1. Nair, H, Allen, M, Anbar, A. D, & Yung, Y. L. A photochemical model of the martian atmoshpere *Icarus* **1994**, *111, 1*,124.

© 2013 by the authors; licensee MDPI, Basel, Switzerland. This article is an open access article distributed under the terms and conditions of the Creative Commons Attribution license (http://creativecommons.org/licenses/by/3.0/).
